# Street play, wellbeing and mental health from childhood into adulthood

**DOI:** 10.1192/j.eurpsy.2023.2107

**Published:** 2023-07-19

**Authors:** R. Nesbit, L. FitzGibbon, H. Dodd

**Affiliations:** 1 Exeter Medical School, University of Exeter, Exeter; 2 Department of Psychology, University of Stirling, Stirling, United Kingdom

## Abstract

**Introduction:**

There is growing interest in the role of children’s play in supporting children’s mental health. Children’s opportunitities for play vary according to the space available to them for play, with more adventurous play happening outdoors (Dodd et al., 2021). The area close to children’s home, such as the street outside their home, can provide an important play space but, with increasing traffic, there has been a decrease in the use of local streets for play over the past generation or two. At the same time we have seen significant increases in children’s mental health problems.

**Objectives:**

Our objectives were: 1) to examine how trends in street play have changed over time; 2) to examine how children’s self-reported wellbeing is associated with their use of streets for play; 3) to examine how adult mental health is associated with their childhood street play experiences.

**Methods:**

Working in collaboration with Save the Children and Play England, we surveyed a nationally representative sample of 1000 children and young people aged 6-16 years, 1000 adults aged 18+ and 1000 parents of children aged 6-16 years about children’s play in their local area and their memories of play during their own childhood. All participants also completed measures of wellbeing (children) or mental health (adults). Participants were recruited from Great Britian.

**Results:**

We found striking differences in the use of streets and local areas for play. For example, across all adults, 62% told us that they regularly played out in their street or area close to home as a child. In contrast 27% of children told us that they regularly play out now. By breaking down the proportion of participants who said they regularly played in their street or local area by their age group, their is a clear decline in outdoor street play over the past 70 years. We asked children about their wellbeing using the Stirling Children’s Wellbeing Scale. Children who said that they regularly played out in their street had higher levels of positive emotion but this was only true for children under 13 years. Adults who told us that they regularly played out in their street or area close to their home as a child had better mental health as adults (lower scores on the K6). Similarly, adults who told us that there was freedom for children to go and explore in the neighbourhood they lived in as a child had better mental health as adults.

**Conclusions:**

Taken together these findings suggest that being able to play in the street or area close to home is linked to wellbeing during childhood and early adolescence and further, that having these expriences during childhood may be beneficial for mental health into adulthood. This indicates that when considering how to support the development of good mental health we should keep in mind the importance of children’s opportunities for play and the role that access to their local enviornment plays in supporting play.

**Disclosure of Interest:**

None Declared

